# Supplementary material for: Distribution and characteristics of microplastics in beach sand near the outlet of a major reservoir in north Mississippi, USA
Source: Microplast nanoplast. Author manuscript; Available in PMC 2024 Jun 21. (PMC11192453; doi:10.1186/s43591-022-00029-z)
Supplement: Supplementary Information — Table S1. Microplastics (1-5 mm size fraction) abundances and morphologies in the beach sand. No microplastics in this size class were found in-between the wrack zones. Table S2. Microplastics (<1 mm size fraction) abundances and morphologies in the beach sand. Figure S1. USGS river gauge data showing the elevation of lower Sardis Lake where the beach is situated, as well as the sampling date (green arrow) and the elevation and date the two wrack zones were formed (red arrows). The lake discharge rates corresponding to the upper and lower wrack zones were ~160 m3/sec and ~125 m3/sec, respectively. Figure S2. Color distribution of small MPs extracted from lower wrack zone (left), in-between zone (middle) and upper wrack zone (right). [file NIHMS1947707-supplement-Supplementary_Information.docx]

**Distribution and characteristics of microplastics in beach sand near the outlet of a major reservoir in north Mississippi, USA**

Zhiqiang Gao,^a^ Kendall Wontor,^a^ James V. Cizdziel ^a,^*, Haitao Lu^b^

^a^ Department of Chemistry and Biochemistry, University of Mississippi, University, MS 38677, USA

^b^ South China Institute of Environmental Science, Ministry of Environmental Protection, Guangzhou 510535, China

Z. Gao and K. Wontor are co-first authors.

* Corresponding author; James Cizdziel

Email: [cizdziel@olemiss.edu](mailto:cizdziel@olemiss.edu)

Tel: 662-915-1814

**Abstract**

Plastic debris both affects and is affected by the beaches it accumulates on. Most studies of microplastics (MPs) in beach sand are focused on coastal beaches or beaches of large lakes near population centers. Here, we assessed MP pollution at a sandy beach near the outlet of a major flood control reservoir (Sardis Lake) in a relatively unpopulated area in north Mississippi, USA, focusing on two prominent wrack zones and areas in-between. Putative MPs were isolated by density separation and matrix digestion, and then examined using stereomicroscopy, with a subset of samples additionally analyzed by µ-FTIR. MP abundance (particles/kg ± 1 standard error (SE), n=15) averaged of 590 ± 360, with 950 ± 100 in the lower wrack zone, 540 ± 40 in the upper wrack zone, and 270 ± 30 in areas between; these differences were statistically significant (*p*<0.01). The MPs generally had similar size and shape characteristics across sites. The majority were fibers (64%), followed by fragments (23%), beads (7%) and films (6%), with a slightly greater proportion of fibers in the wrack zones compared to areas in-between. The number of MPs rose dramatically with decreasing size. Beads were only found in the <500 µm size fraction. Clear and blue were the predominant colors for all MPs. A total of 29 different types of polymers were detected, with more than half of the particles being composed of polyethylene and polyamide, followed by poly(methyl methacrylate), polyethylene terephthalate, polycarbonate, polypropylene, and others; although this distribution varied some depending on size fraction and location. Because there are no major wastewater discharges into Sardis Lake, the source of the MPs is likely degradation of carelessly discarded plastic, as well as atmospheric fallout. Overall, we found that MP concentrations were highest in the wrack zones and influenced by rates and duration of discharge from the reservoir. Thus, like coastal beaches, wrack zones on freshwater beaches along or downstream of reservoirs accumulate both macro- and micro-plastics and are prime locations for plastic cleanup. Finally, we show that MPs made from naturally weathered LDPE plastic film are prone to fragmentation during pretreatment procedures, which may result in its overestimation.

**Keywords:** Microplastics; Beach sand; Wrack zone; Reservoir; Sardis Lake; Density separation; Stereomicroscopy; µ-FTIR; Weathered microplastics; Recovery experiment

**Introduction**

The explosion of microplastics (MPs) research in the past few years has led to a greater understanding of just how widespread these contaminants are. MPs have been found in food^1,2^, soil^3^, water^4^, and air^5^ in both urban environments^6^ and far more remote locations^7^. Recent research has even shown that the average human adult ingests hundreds of MP particles per day.^8^ Such studies and potential exposures to both humans and wildlife indicate the need to further characterize the occurrence, distribution, sources and risk of these widespread contaminants. Usually defined as plastic particles smaller than 5 mm,^9^ MPs can be categorized in a variety of ways. For example, MPs manufactured below 5 mm in size are defined as primary MPs and those resulting from the breakdown of larger plastic products defined as secondary. Additionally, MPs are often categorized based on morphology, i.e., fiber, fragment, film, or bead, as well as chemical composition.

Early research into MPs mainly focused on their presence in marine waters. As a result, a vast amount of literature has been published on MP levels in the oceans, particularly along coastlines.^7,10–12^ There is also a growing body of research on MP pollution in freshwater systems around the world, representing another potential exposure route for humans and wildlife.^13–16^ More recently, researchers have examined the deposition of MPs from natural waters into sediment, especially beach sand. One reason for this focus is that MPs are more prone to both mechanical and, to an extent, UV degradation in sand than in water.^17^ Increased exposure to these breakdown pathways can therefore result in both larger quantities and smaller sizes of MPs. This is concerning because research has shown that MPs’ toxicity may increase as their size decreases.^18^

In addition to these potential health effects, MPs in sand could result in environmental changes as well. The presence of MPs has been shown to increase sand permeability, allowing water to flow through more easily.^19^ It is also deemed to affect the temperature of the sand, decreasing both the rate of heat flow and the ultimate sand temperature. This in turn might have a large effect on coastal and marine animals, particularly sea turtles, for whom sex determination depends on the temperature of the sand their egg is nested in, as well as on meiofauna, present in both marine and freshwater beaches.^19^ These temperature and soil permeability changes could also have an impact on the makeup of the microbial community of the sand, leading to changes in the levels of carbon and nitrogen cycling performed by these microorganisms.^20^ Such microbial changes may result in increased human health risks, as beachgoers can be exposed to MPs that act as colonization sites for *Vibrio spp*. and *E. coli*.^21^

Given these potential human health and environmental effects, many studies have characterized MPs in beach sand, with most focused on coastal beaches^22–26^ and, on occasion, beaches of large lakes.^27,28^ However, there is a current lack of research on MPs in beach sand at freshwater reservoirs. Such reservoirs represent important water sources to the communities around them, with their beaches additionally acting as popular recreation sites. This high public use in conjunction with the potential negative effects of MPs listed earlier makes analysis of such areas important.

The aim of this study was therefore to quantify and characterize MPs present in beach sand near the outlet of one such reservoir, Sardis Lake in northern Mississippi. A secondary aim was to evaluate recoveries for extraction procedures using MPs generated from naturally weathered plastics, which are more realistic than what is typically employed such as virgin plastics beads direct from the manufacturer. To our knowledge, this is the first study on MPs in sand from a beach located near the outlet of a major flood control reservoir, the source of much of the plastic.

**Materials and Methods**

***Study area***

Sardis Lake is one of four major flood control reservoirs, created with earth fill dams, in the upper Yazoo River Basin in north central Mississippi (Figure 1). It has a watershed of ~3985 km^2^ and a summer pool of ~130 km^2^. The water levels are regulated to help control flooding in the agriculturally important Mississippi Delta, with a fall and winter drawdown to capture high spring rainfall and maintain a summer recreation pool. The lake is extensively used for recreation by the public, and is especially popular for crappie fishing. Sources of plastic to the beach include carelessly discarded plastic by beachgoers (primarily during the summer), upstream fishers (anglers along the shore of the outlet channel), and plastics that flow through the intake structure on the upper lake entering the lower lake via the outlet channel. The intake gates for discharging water from the reservoir are at an elevation of ~70 m, but their depth below the surface of the lake varies between 1 to 10 m, depending on rainfall, season, and other factors as described earlier.


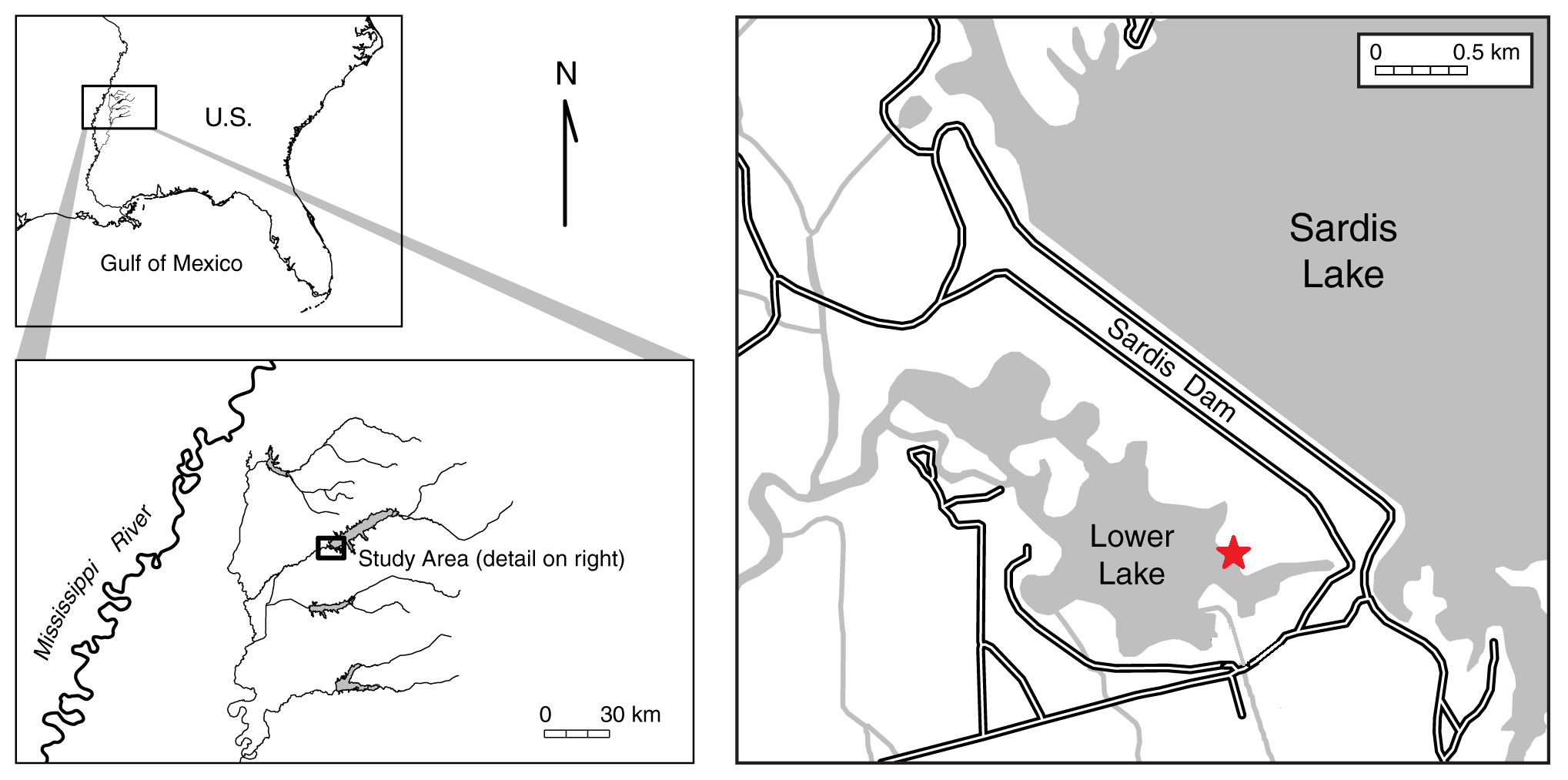


**Figure 1. Maps showing the study area in north Mississippi. Samples were collected from a sandy beach (red star) located in the lower lake ~0.5 km from the outlet (discharge) channel of Sardis Lake. Photos of the sampling site are provided in Figure 2.**

***Sample collection.***

Samples were collected following a NOAA protocol outlined in MPs sampling and processing guidebook from Mississippi State University.^29^ Briefly, beach sand was collected from two different wrack zones as well as areas in-between. Wrack zones consist of an accumulation of debris deposited at high water levels, usually high tide, but here associated with discharge flows through the dam outlet channel regulated by the US Army Corps of Engineers. We sampled at five different locations in each of the three zones using a 0.25 m^2^ quadrat (Figure 2). Large pieces of natural debris (e.g., sticks, feathers) were removed after shaking them free of sand. A metal scoop was used to remove the top 3 cm of sand in the quadrat, which was then placed into a large zipper-seal polyethylene bag and labelled.


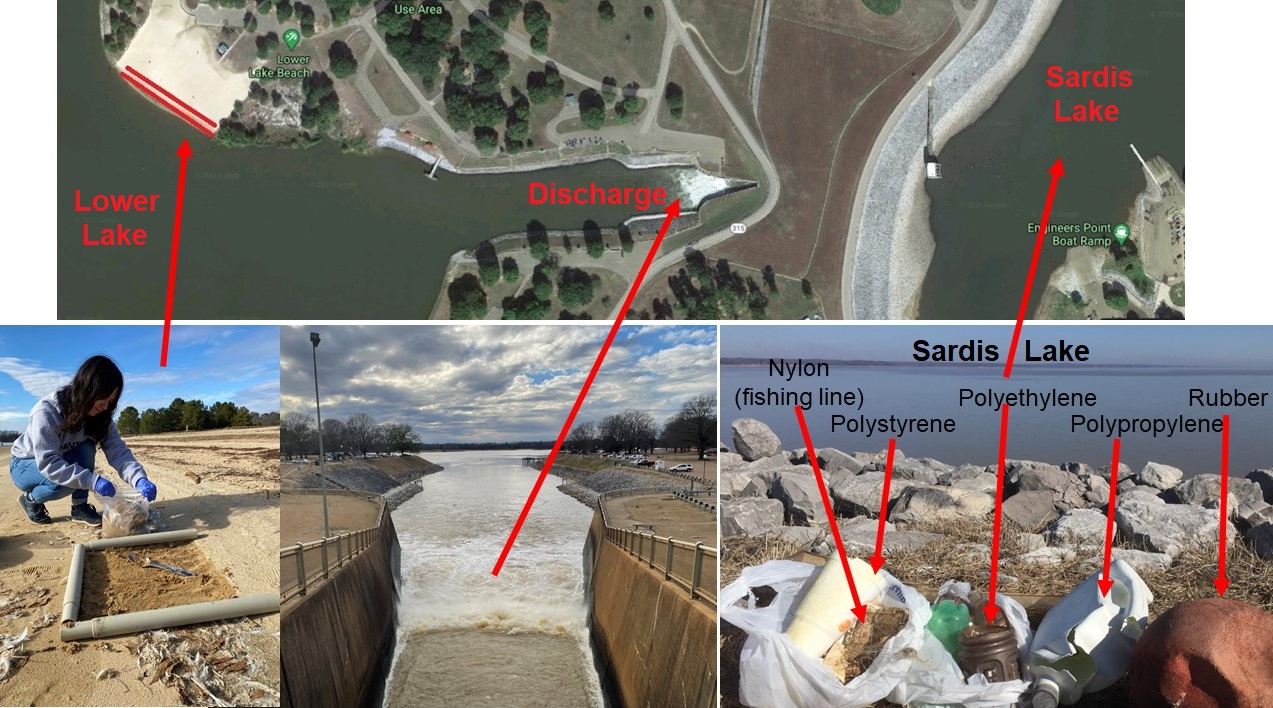


**Figure 2. Aerial view of the study site (top) showing the beach with wrack zones (red lines), discharge from the reservoir to the lower lake, and earthen dam creating Sardis Lake. Photos show sampling quadrats of the beach sand (lower left), view of discharge into the lower lake (lower center), and plastic debris recovered from upper Sardis Lake (lower right).**

***Drying and sieving of sand samples***

The beach sand was transferred into metal pans and placed in an oven where it was dried at 60°C for 3 days. The samples were then passed through 5-mm and 1-mm stainless steel sieves, and each size fraction was placed in a zipper-sealed polyethylene bag and stored in a drawer until further processing. A blank study determined that the bags did not contaminant the samples.

In retrospect drying the samples at 60°C was a mistake as the temperature is at or exceeds the glass transition temperatures for some forms of nylon, including nylon 6 and nylon 12. This is problematic because it can denature the polymer which in turn can lead to difficulty in identifying the polymer by FTIR. Thus, our results may underestimate the abundance of nylon in the sample. Other polymers in this study were not affected.

***Isolating microplastics by density separation and digestion of natural organic matter***

MPs were extracted from the beach sand using a density separation as described elsewhere.^30^ Briefly, ~200 mL of ZnCl_2_ solution (certified ACS grade, purity>99%, Fisher scientific) with a density of 1.6 g/mL was added to 100 g of sand in a 250 mL glass beaker. The mixture was stirred with a glass rod for 1 min and then allowed to settle for 6 h. The top (floating) layer of debris was transferred into a glass vial using a 5 mL glass pipette. This process was repeated twice more. The collected floating debris was re-sieved through either a 45 µm mesh screen for sand samples <1 mm or a 1 mm mesh screen for sand samples 1 to 5 mm in size and rinsed with 1% HCl solution and MilliQ water. The particles on the screens were then transferred to 250 mL clean glass jars by rinsing with milli-Q water passed through a 47 mm (0.2 µm pore size) polycarbonate track-etched filter (Sterlitech Corp., Kent, WA, USA). Especially dense polymers (e.g., polytetrafluoroethylene) were not included/targeted in this study.

A matrix digestion was needed because natural organic matter in the samples (e.g., bits of leaves and feathers) also floated to the surface during the density separation. We attempted to remove this natural organic matter using Fenton’s Reagent, but found that it did not fully digest it. Thus, we employed an alkaline digestion by adding 50 mL of 10% w/v KOH solution to the extracted material and left it covered at room temperature overnight. The next day the digests were filtered through 47 mm polycarbonate filters for visual inspection by stereomicroscopy.

It is important to note that KOH treatment at elevated temperatures can alter the appearance (and FTIR spectra) of some types of MPs, such as nylon 6, nylon 66, PET and polycarbonate.^31,32^ It is best to apply “soft” pretreatment procedures whenever possible, avoiding strong reagents and elevated temperatures, to accurately quantify the abundance of MPs in samples.

***Stereomicroscopy and selection of putative microplastics***

Each extract was visually inspected at 70× magnification using a Stemi 508 stereomicroscope (Carl Zeiss, Jena, Germany) equipped with an Axiocam 105 color digital camera and an X-Cite 120Q fluorescence lamp illuminators. Synthetic fibers were distinguished from non-plastic fibers and natural plastics based on published criteria recommending inclusion of particles lacking distinguishable cellular or biological structures, objects with even coloring, and fibers with uniform thickness.^33^ Putative MP particles were then classified by both morphology and color. Morphology was categorized as either a fiber, fragment, bead or film.^34–36^ Colors were designated as either clear, blue/green, black, brown, red/pink, yellow, white or purple. Representative MPs were photographed and the major dimension of each particle was measured. All possible MPs were enumerated and fluorescent particles in blanks were counted under red fluorescence range (excitation at 560/40 nm, emission at 620/40 nm).

***µ-FTIR analysis***

To identify MPs, putative MPs on 47 mm filters were transferred to 5 mL 50% ethanol via sonication. 2 mL aliquots were filtered through 25 mm gold-coated polycarbonate track-etched filters (25 mm diameter, 0.4 µm pore size; Sterlitech Corp., Kent, WA, USA). Eight 1 mm × 1 mm sections were randomly selected on each filter and analyzed by µ-FTIR imaging using a Perkin Elmer Spotlight 200i. Measurements were conducted in in the reflectance mode using a mercury cadmium telluride (MCT) detector. Spectra were taken at 24 scans with wavelengths between 600 and 4000 cm^-1^ and a spectral resolution of 4 cm^-1^. Sample spectra were compared to the spectra library supplied by Perkin Elmer; matches were deemed positive with >70% similitude between sample and library spectra.^37^

***Contamination mitigation, blanks, and spikes***

Cotton laboratory coats and nitrile gloves were worn during sample preparation and analyses. Glassware was thoroughly rinsed and/or heated at 450°C for 4 hours, plastic materials were maximally avoided, and samples were kept covered or sealed unless being actively processed. Sample preparation procedures were conducted in a clean room to reduce airborne contamination. Twelve blanks were analyzed each consisting of 100 g of pure sand (EMD Millipore) heated to 450°C for 4 hours. To assess recovery, we spiked six of these blanks with MPs of different sizes and morphologies. Three blanks were spiked with 20 fluorescent PE beads (Cospheric, CA, USA), 10 beads ranging in size from 150-180 µm and 10 from 300-355 µm, as well as with 10 fragments of PMMA ranging from 500-1000 µm. The PMMA particles were cryogenically ground (SPEX freezer mill) and stained with pink synthetic dye (Rit DyeMore, Nakoma Products LLC). Three other blanks were spiked with 10 stained particles (1-5 mm in size) of weathered LDPE film, also cryogenically ground, and 10 stained polyester fibers (1-5 mm) cut with a scissor. The remaining six blanks were not spiked. Each of these 12 samples was processed in the same manner as the beach samples.

**Results and Discussion**

***Contamination and method validation***

Despite stringent controls we found MPs in all our blanks, demonstrating the need to include multiple blanks in all MP studies. Here, the blanks averaged 63 ± 24 particles/kg, amounting to ~10% of the average for the beach sand. All sample data herein was blank subtracted according to morphology. Most of the MPs in the blank samples were either fibers (86.2%) or fragments (12.1%). Most of the MPs found in the blanks were PEST (28.6%) or PE (14.3%), both common contaminants, with PEST often associated with synthetic fibers from clothing. There was no significant relationship between the major dimension of MPs in blanks and sand samples (*p*>0.05).

We spiked clean sand with several morphologies and polymer types of MPs to assess recoveries from sample preparation. Recoveries for spiked samples ranged from 76.7% to 310% (Table 1). The lowest recoveries were for PEST fibers, possibly because they are prone to waft into the air, stick to the walls of glass equipment, and can sometimes pass through sieves with mesh sizes smaller than the fibers length if oriented properly. The highest recovery was for LDPE films from weathered (brittle) wrappers, which were cryogenically ground into MPs. The shape of the film MPs (flat and thin) seemed prone to fragmentation during sampler preparation (Figure 3B). However, the resulting fragments were still film-like and thus would not contribute to other morphological categories. The other spiked MPs, including fluorescent PE beads, polyester fibers, and weathered PMMA particles, were observed to remain intact during sample preparation (Figure 3A, C). Because our recoveries, except for the film category, were generally good and because the spiked materials were not representative of all plastics (e.g., types, shapes, sizes), we do not correct for recoveries herein. Also, while the abundance of film MPs in the sand samples may be somewhat overestimated (based on our high recoveries), they still represent a small portion (<8%) of the overall MPs measured.

| **Table 1. Results for recovery experiments (n=3 in each category).** | | | | | |
| --- | --- | --- | --- | --- | --- |
| Shape | Type | Size Range | Stained | Source | Average Recovery (%) |
| Beads | PE | 150-180 µm | No, already fluorescent | Cospheric, CA, USA | 83.3 |
|  |  | 300-355 µm |  |  | 96.7 |
| Fragments | PMMA | 500-1000 µm | Yes | Weathered plastic from Sardis Lake | 100 |
| Films | LDPE | 1-5 mm | Yes | Weathered plastic from Sardis Lake | 310 |
| Fibers | PEST | 1-5 mm | Yes | Univ. Hawaii Polymer Kit 1.0 | 76.7 |

| 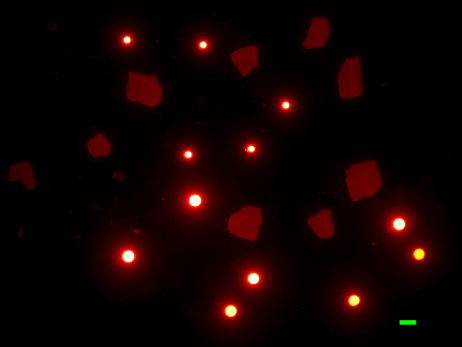  **A** | 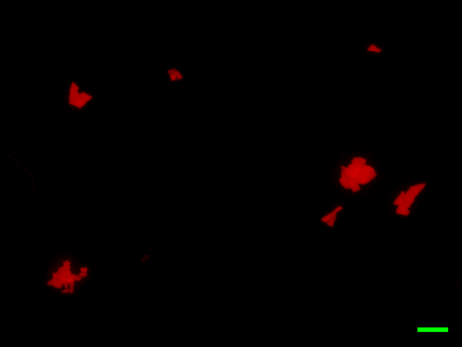  **B** | 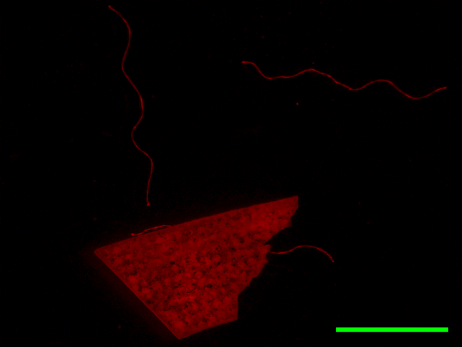  **C** |
| --- | --- | --- |

**Figure 3. Images of fluorescent microplastics used in recovery tests: (A) Two size fractions of beads (150-180 µm and 300-355 µm) and stained PMMA fragments (500-1000 µm); (B) Weathered LDPE film fragments (~50-500 µm); (C) Stained films and polyester fibers (~1-5 mm). Green scale bars represent 500 μm (A and B) and 2 mm (C).**

***Abundances of microplastics in the beach sand***

Comparing beach MP data from literature studies is problematic due to non-standardized sampling, extraction, and analysis methods, along with different units used to report results (particles/kg or particles/m^2^).^38,39^ Here, we focus our discussion using particles/kg, but include data in particles/m^2^ in Tables S1, S2. On average, concentrations of MPs were 950 ± 100 particles/kg in the lower wrack zone, 540 ± 40 in the upper wrack zone, and 270 ± 30 in areas in-between (Figure 4). Microplastics in the largest size fraction (1–5 mm) were found in both the lower and upper wrack zones, but not in-between, with the lower wrack zone having 60 ± 41 particles/kg and the upper wrack zone 30 ± 26 particles/kg (Table S1). The abundance of small MPs (<1 mm) in the sand from the three zones averaged 560 ± 330, ranging from 270 ± 110 in between the wrack zones to 890 ± 340 in the lower wrack zone (Table S2). Overall, these smaller MPs were 19 times more abundant than the larger size fraction. The lower wrack zone had statistically higher MP concentrations (average 885 ± 336 particles/kg) than the upper wrack zone (average 510 ± 140 particles/kg) (*p*=0.002) and areas in-between (average 270 ± 110) (*p*<0.001). As expected, concentrations in both lower and upper wrack zones were higher (*p*<0.01) than the sand collected in-between them. Sand collected from lower wrack zone also showed higher variation of MP concentrations than the upper zone (*p*=0.670) and in-between zone (*p*=0.318).


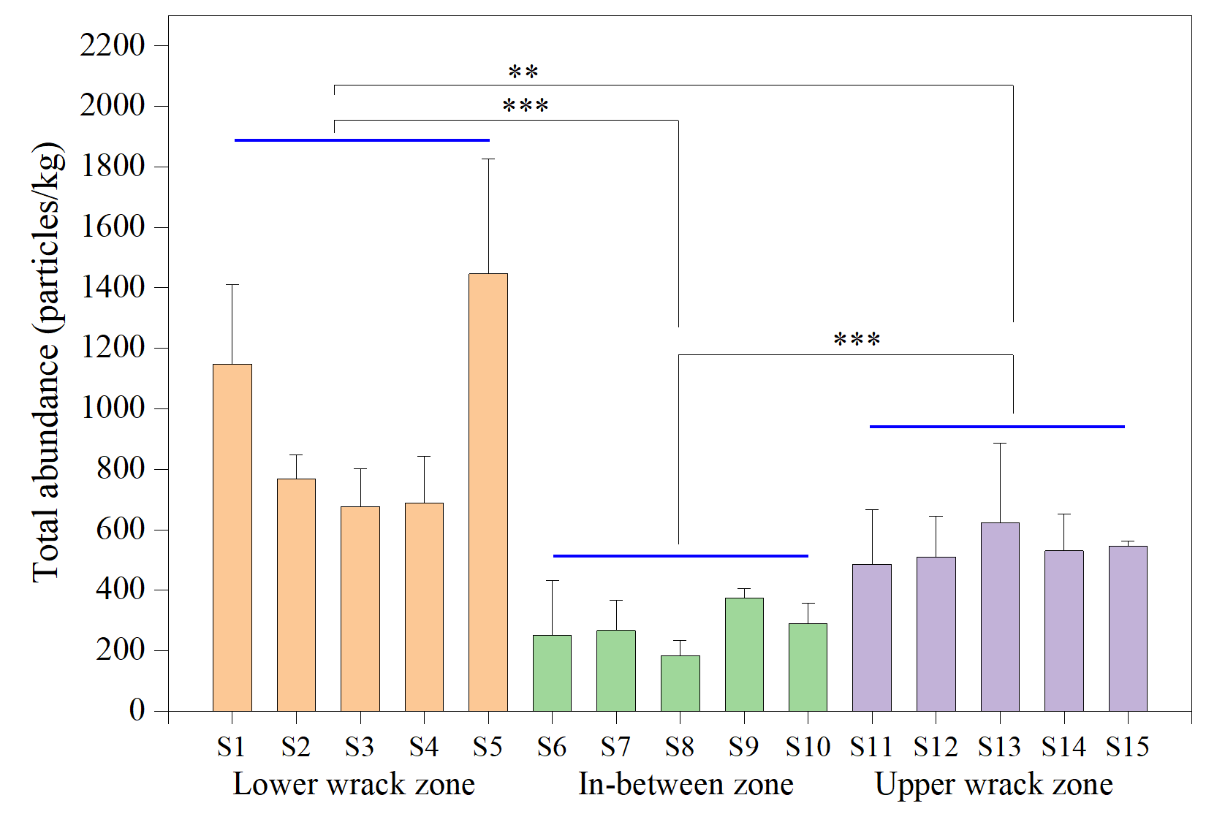


**Figure 4. MP abundances ± 1 standard deviation (n=3) in beach sand collected from five quadrats along two wrack zones and in-between zone near the outlet of Sardis Lake. ** represents *p*<0.01; *** stands for *p*<0.001.**

There are a several possible reasons for these spatial differences. We have previously shown that flooding in the Mississippi River tends to decrease the concentration of MPs in the water; although flooding may increase the overall plastic load, the excess water likely results in a dilution effect.^40,41^ This dilution effect is similarly present in Sardis Lake during times of high lake volumes. In reservoirs, high lake volumes often precede periods of rapid lake discharge, leading to the formation of the upper wrack zone. In contrast, the lower wrack zone develops over longer periods during lower and more stable discharge rates. Further, because lower wrack zones can be washed free of debris during high discharge rates, lower zones are newer than the upper wrack zone. Examining the lower lake elevation and upper lake discharge rates prior to sampling (Figure S1) suggests that this scenario is likely the case here. Indeed, the slope of the decline in lake elevation during the last high discharge period is greater than during the formation of the lower wrack zone. Thus, it is not surprising that the upper wrack zone exhibited lower concentrations of MPs compared to the lower wrack zone.

Despite the difficulty in comparing results, average concentrations of MPs at lower Sardis Lake beach fall within the range observed for beaches globally. At the lower end of this spectrum, a study of beaches in northwestern Mexico found an average of 140 ± 90 particles/kg, with over half the sites showing concentrations below 100 particles/kg.^42^ At the higher end of the scale, the average concentration in four protected beaches from barrier islands in Virginia and North Carolina was 1410 ± 810 particles/kg.^43^ Our findings of an average of 590 ± 360 particles/kg are therefore roughly in the middle range. It should also be noted that it is not uncommon for there to be large variations in MP concentrations not just between beaches, but within the same beach.^44^ Our study also shows relatively large variations can occur in different sites on a single beach, thus demonstrating the need to sample at multiple sites throughout the beach, including wrack zones.

***Physical characteristics of the microplastics in the beach sand***

Physical characteristics, including morphologies, sizes and colors, may enable researchers to track the sources of MPs.^45,46^ Here, representative images of MPs obtained by stereomicroscopy are shown in Figure 5. Characteristics for the larger size fraction of MPs (1-5 mm) were consistent between wrack zones, which were dominated by fibers, fragments, and films (Table S1). We did not find MPs in this size class for samples collected in-between the wrack zones. Smaller MPs (<1 mm) also were relatively consistent across the beach with similar shape characteristics and size distributions (Table S2; Figure 6). The majority of these were fibers (61%), followed by fragments (25%), beads (8%), and films (6%). Other studies have also found fibers as the dominant form of MPs in beach sand.^27,43^ The high number of fibers are often associated with the discharge of wastewater, not the breakdown of plastic debris.^38,47^ However, because there are no major wastewater discharges to Sardis Lake, our results suggest that other sources, including atmospheric fallout, are contributing to widespread fiber pollution.

| 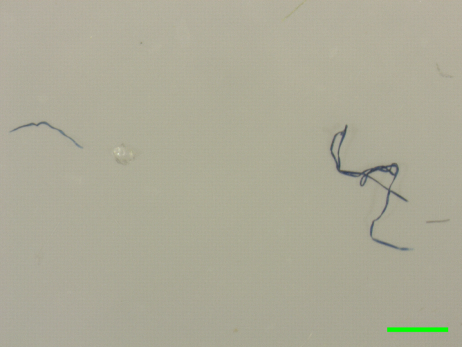  **AC** | 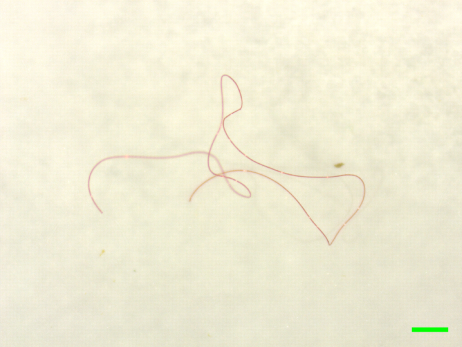  **B** | 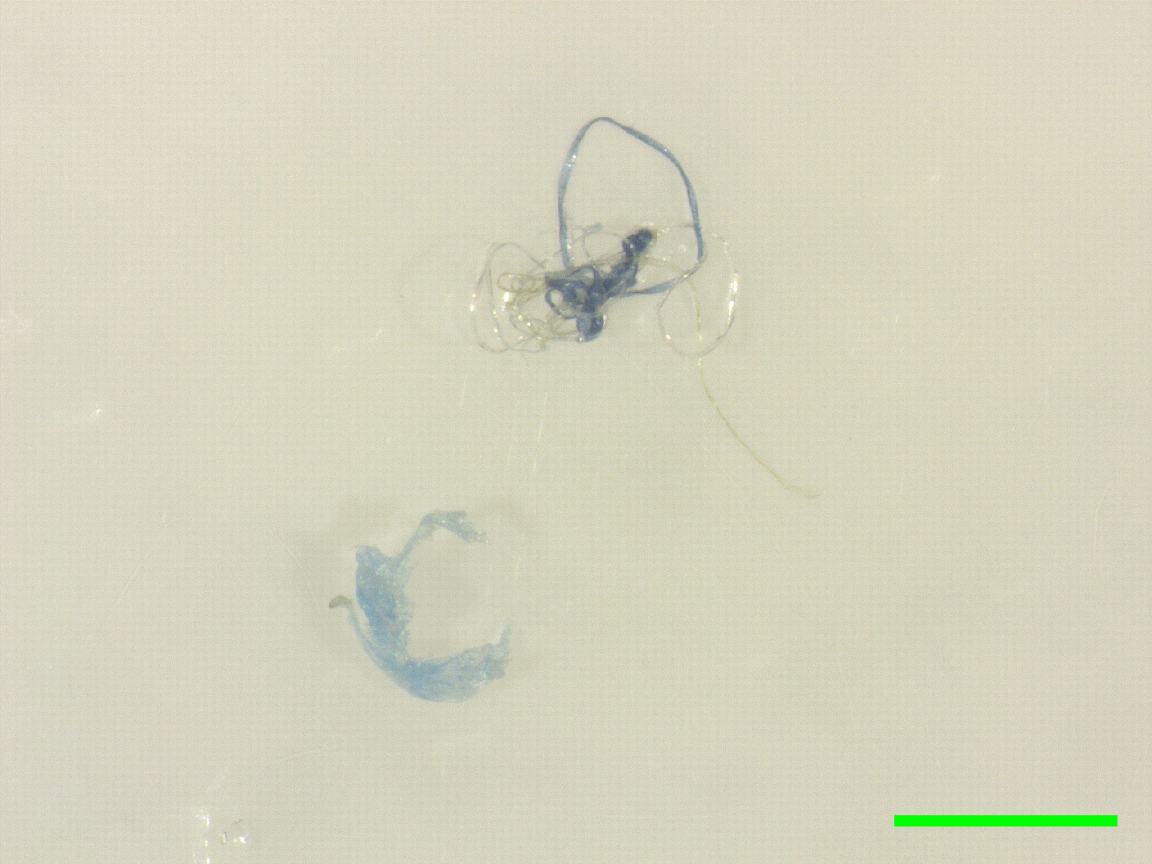  **C** |
| --- | --- | --- |
| 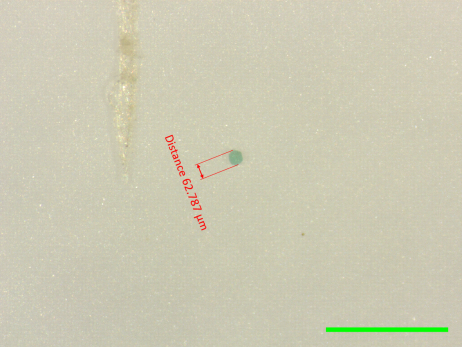  **G**  **D**  **E** | 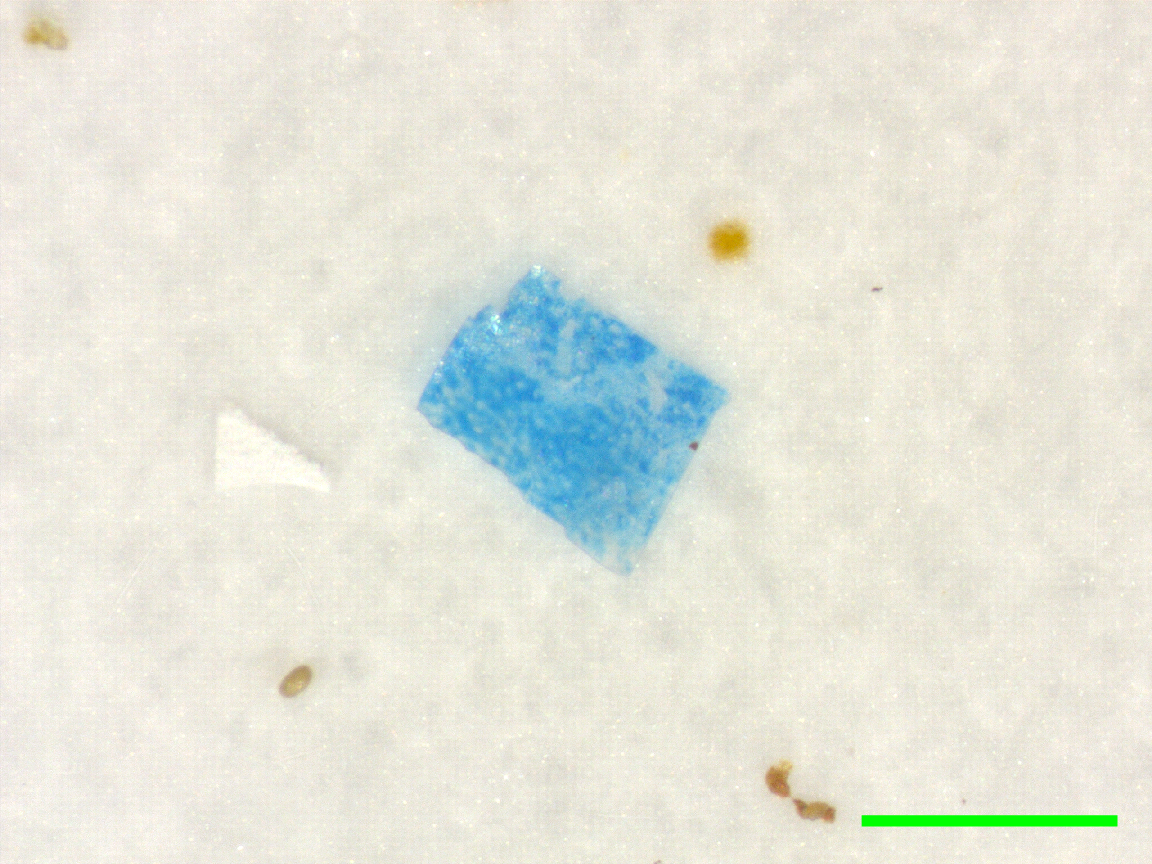  **H**  **F** | 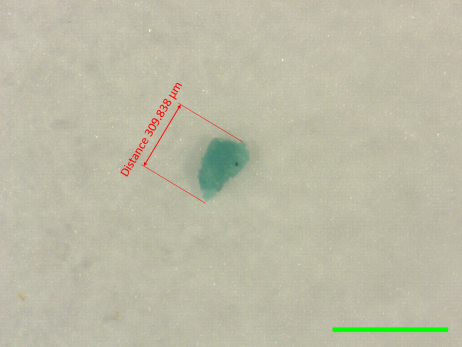  **I** |
| 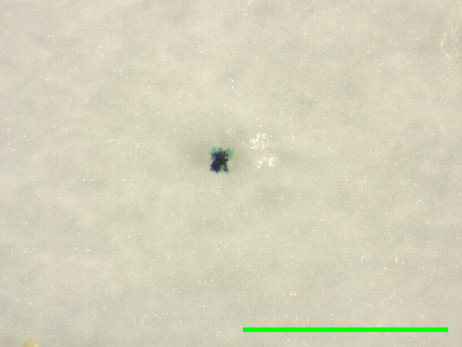 | 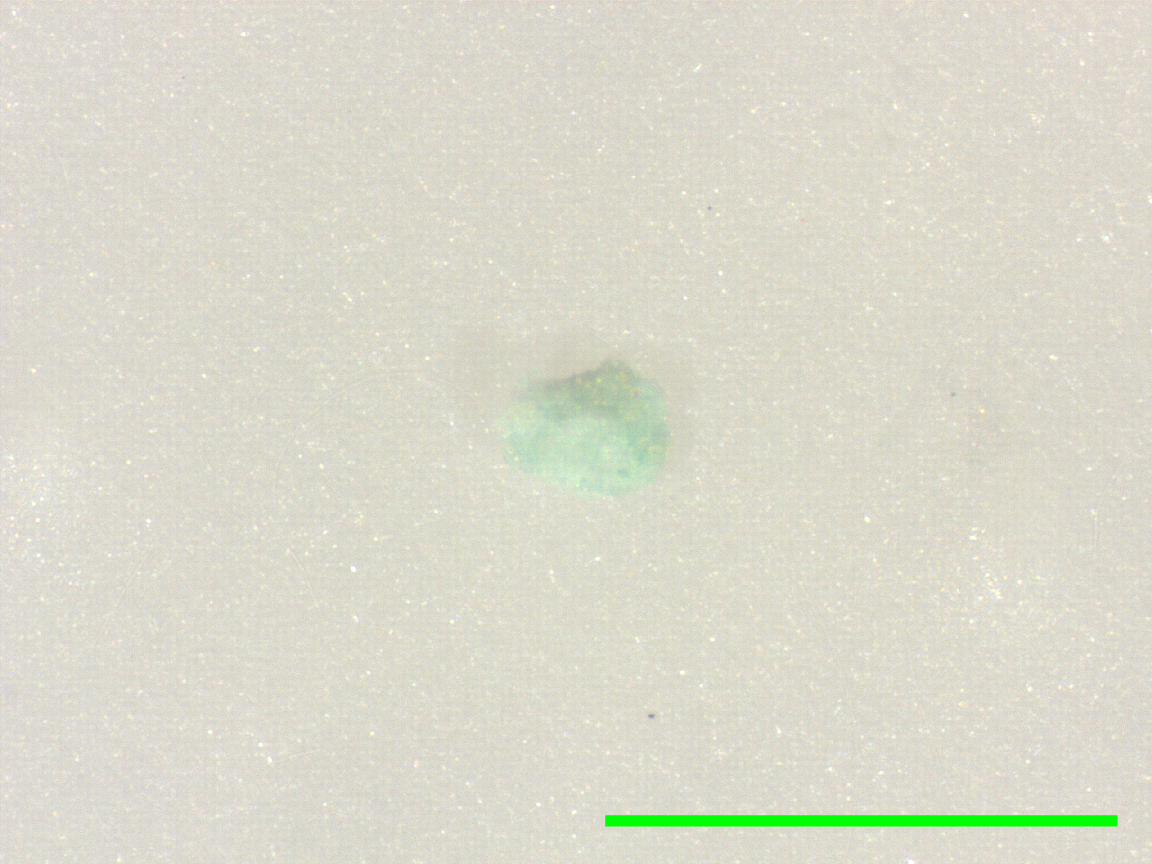 | 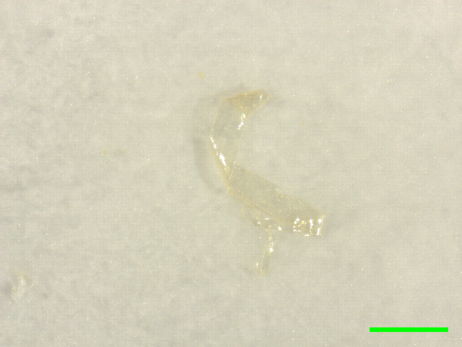 |

**Figure 5. Microphotographs of microplastics from beach sand in lower Sardis Lake: blue fiber (A); long red fiber (B); entangled fibers and blue fragment (C); green bead (D); blue film (E); blue fragment (F); black-blue colored fragment (G); blue fragment (H); and clear fragment (I). Each green scale bar is 500 µm in length.**

| ***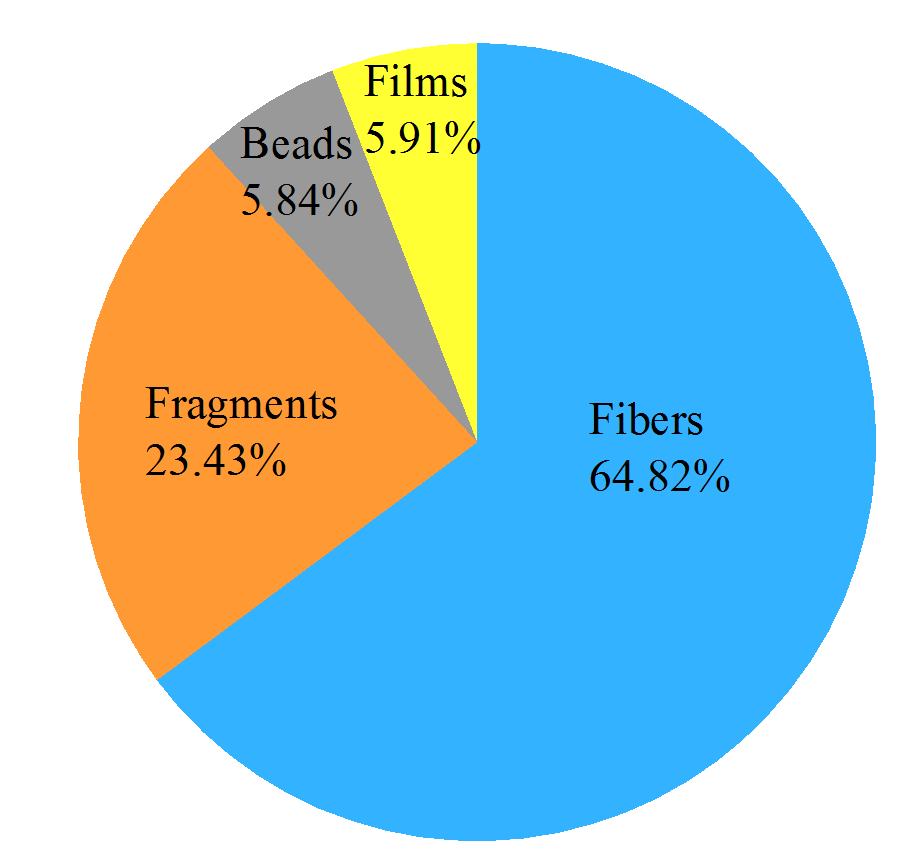*** | ***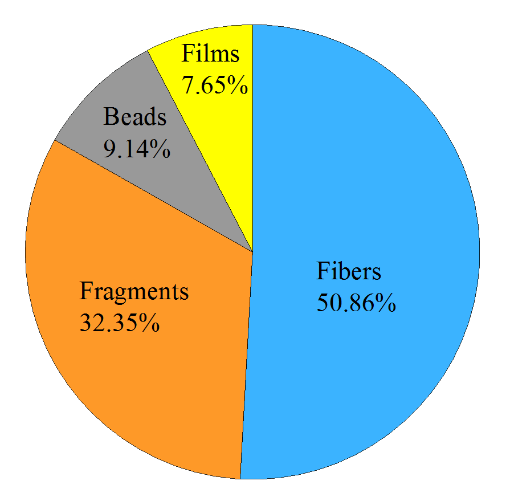*** | ***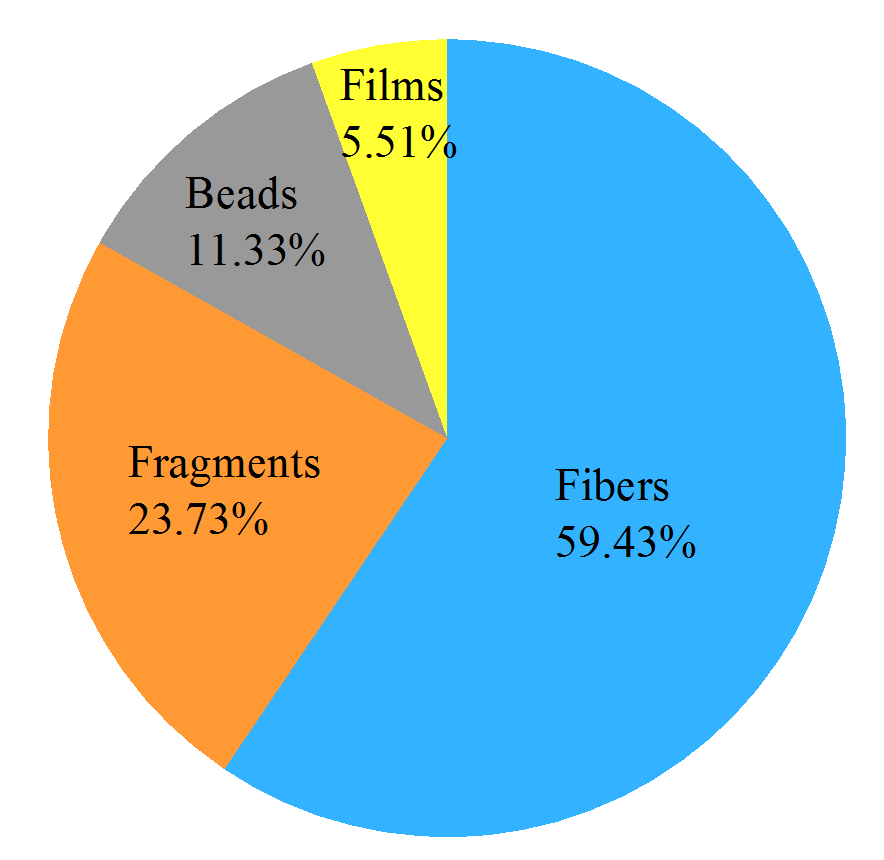*** |
| --- | --- | --- |

**Figure 6. Morphologies of microplastics (<1 mm) in sand from a lower wrack zone (left), an in-between zone (middle) and an upper wrack zone (right) at a beach near the outlet of Sardis Lake.**

Particle size is an important factor in the assessment of MP threats to biota, as smaller MPs can be consumed by both terrestrial and aquatic organisms^31,48,49^ Additionally, these small particles are cause for concern because their relatively large surface to volume ratio makes them capable of adsorbing a wide variety of pollutants.^50,51^ Consistent with other studies,^24,52^ the bulk of MPs in our samples were in the lowest size fractions (Figure 7). Further, the particle size distribution was consistent across the beach with no statistical differences between sites (*p*>0.05). More specifically, fibers had the largest average major dimension (1000 ± 900 µm), followed by films (460 ± 230 µm), fragments (180 ± 140 µm), and beads (70 ± 40 µm). Much of these are likely secondary MPs stemming from degradation of larger plastic debris that will continue to degrade over time.

Several fibers >10 mm in length were found in sand sieved through a 1 mm mesh screen; however, this is not unusual given the fine width of most fibers.^53^ Recognizing that evaluating MP sizes based solely on sieve sizes can be biased, particularly for fibers, we included measuring individual particles through stereomicroscopy. However, size estimation based on sieving remains a common practice for samples with large numbers of MPs (e.g., wastewater, sludge, landfill leachate) given the impracticality of measuring every single MPs.

| 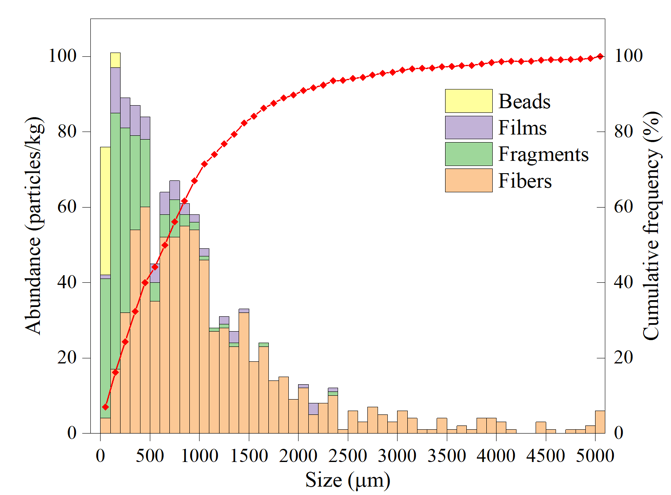  **AC** | 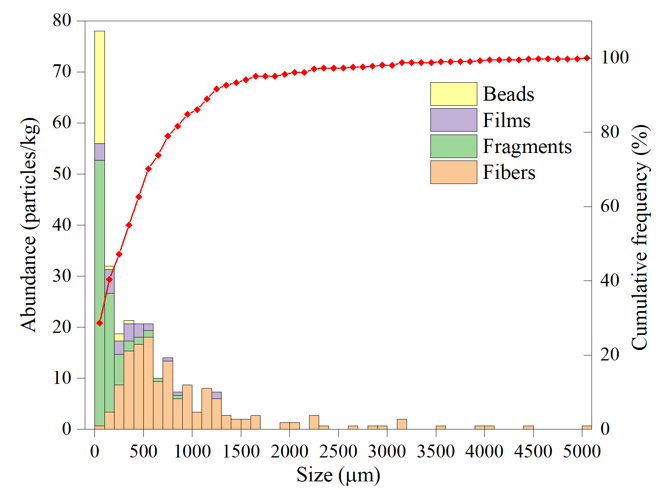  **BC** |
| --- | --- |
| 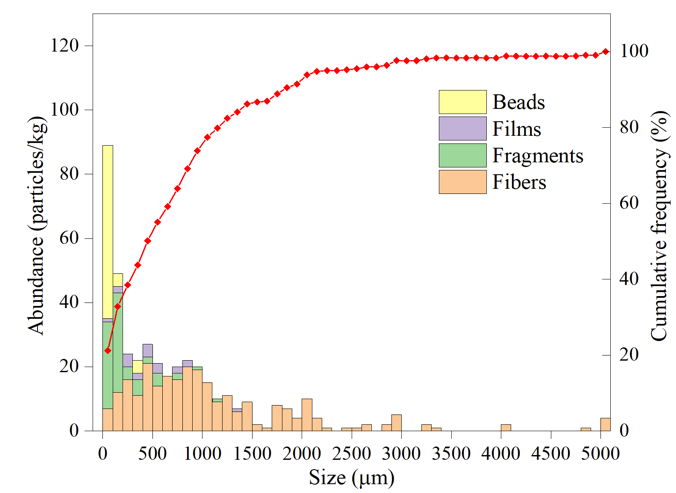  **CC** |  |

**Figure 7. Size distribution of microplastics extracted from lower wrack zone (A), in-between zone (B), and upper wrack zone (C).**

Color is another important feature of MPs in the aquatic environment since marine organisms have been shown to ingest MPs with coloring similar to their prey.^54,55^ Microplastics can be either a single color or a mix of colors.^56,57^ These colors do not come from the plastics themselves, but rather from chemical additives incorporated during the manufacturing process. Previous studies have shown that transparent (clear) and blue are the two most commonly found single colors in environmental MPs.^25,27,58^ In our study, clear and blue particles also predominated (Figure S2). It is worth noting that MPs subjected to natural weathering conditions can become discolored.^32^ As plastics degrade, additives and dyes are able to be leach out from the polymer matrix since they are not usually covalently bonded to the polymer structure.^59^ Similarly, color leaching may occur due to the use of oxidative reagents in certain digestion methods. Both of these may contribute to the high level of transparent MPs in this and other studies.

***Chemical identification of the beach microplastics***

Common polymers detected at marine beaches in other studies were also identified in our samples. Five different polymers were identified in the upper wrack zone for the >1 mm size fraction of MPs. Most were PE (37%), followed by PS (25%), PNB (13%), Polyacrylate:PS (13%) and PET (12%) (Figure 8). PE was also predominant in lower wrack zone (22%), followed by PA (18%), PP (13%), PMA (9%) and PC (9%). Other polymers identified include PET, PMMA, POM, PS, PVDF and copolymers (PE:PB and PVDF:PHFP).

Additional polymer types were detected in smaller size fraction (<1 mm). PE was again predominant across all sites. PE is made into products ranging from clear food wrap and shopping bags to detergent bottles, which are produced and used globally. PS, PP, PEST, PC and PET were also found in both wrack zones. These plastics are widely employed in food-service industry as containers, disposable utensils, and drink holders, and in other household products. Nylon (polyamide) and polyacrylates were also detected. The former is used, for example, as fishing line, and the latter is commonly found in paint and other coatings. Although fibers were the most abundant morphology observed, the proportion of PEST (identified by µ-FTIR) was generally low, suggesting that domestic wastewater, typically laden with PEST fibers, may not be a major source of MPs to Sardis Lake. Other plastics and cellulose can also take on the shape of fibers, including nylon from fishing gear. The distribution of the remaining polymers varied somewhat between sampling sites.

Apart from homopolymers, copolymers were also sporadically detected. Copolymerization is used to modify the properties of manufactured plastic to meet specific needs, for example to reduce crystallinity, modify glass transition temperature or to improve solubility. These copolymers may be used to help identify the source of the MPs they are associated with. Regardless of sampling sites, low-density polymers (e.g., PE, PP, PS, PBMA) were most frequently found. This is potentially because denser polymers tend to locate in lake sediment, whereas low-density polymers are more likely to remain buoyant, which allows them to migrate and deposit on the beach. Additionally, some studies do not observe denser polymers like PET and PVC, because they employ a saturated NaCl solution for MP extraction.^60^ However, we used a solution of ZnCl_2_ with a density of 1.6 g/cm^3^ to extract MPs, which is far more effective for these denser plastics. This suggests our reported concentrations of MPs, including high-density polymers, reflect their true concentration in beach sand and not recovery issues.

| 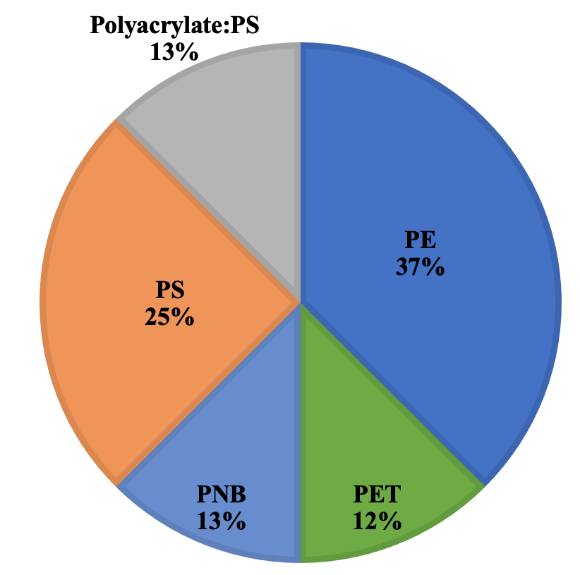  **A** | 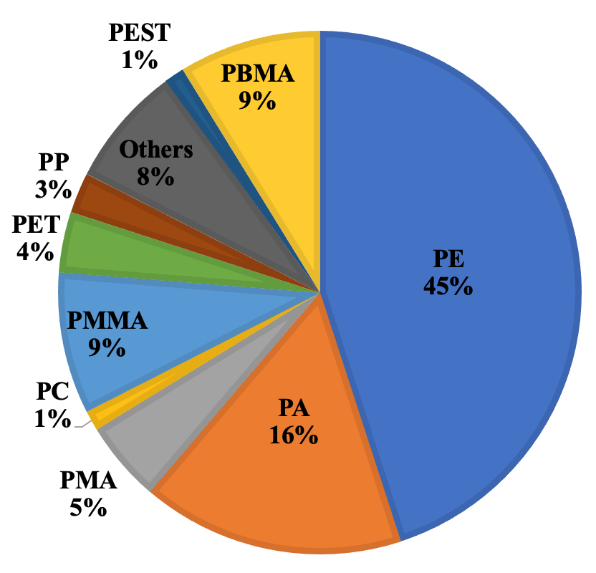  **B** |
| --- | --- |
| 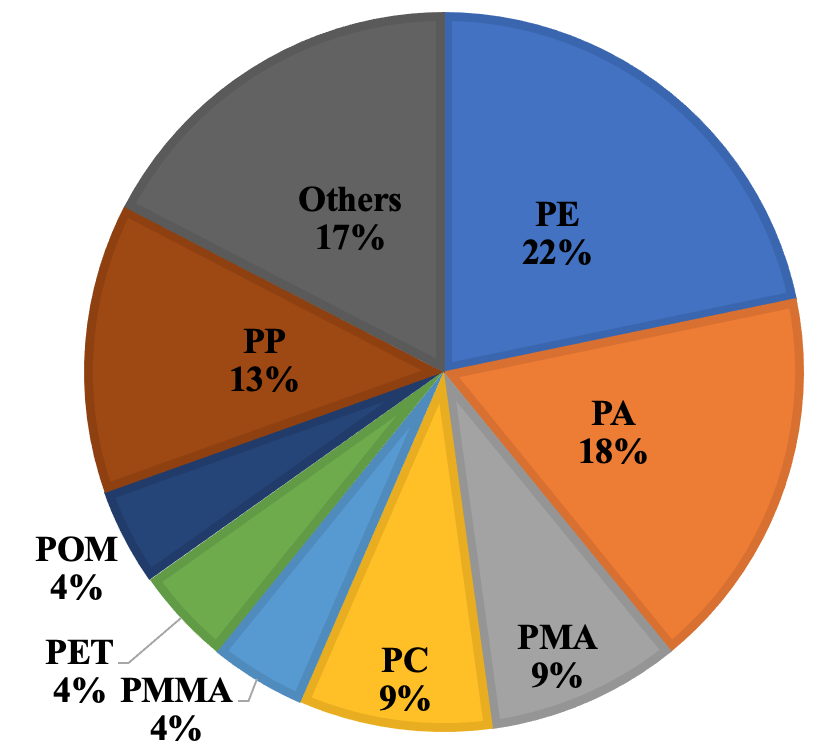  **C** | 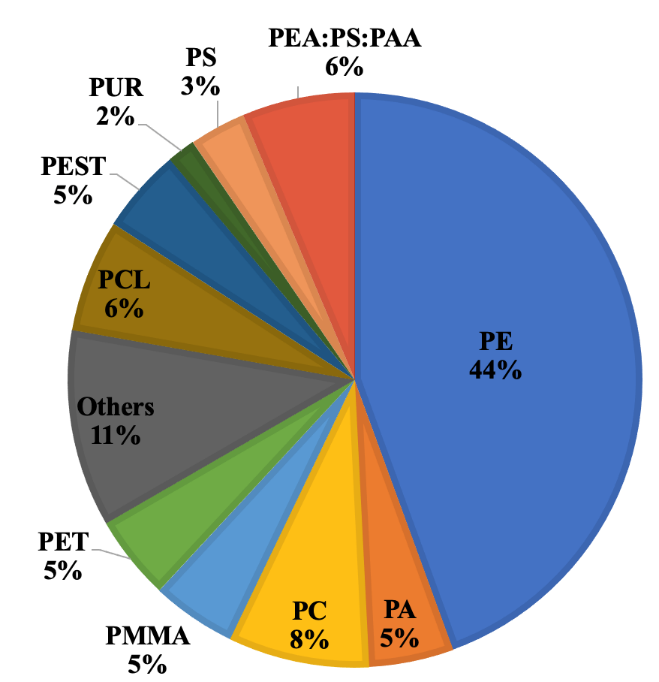  **D** |
| 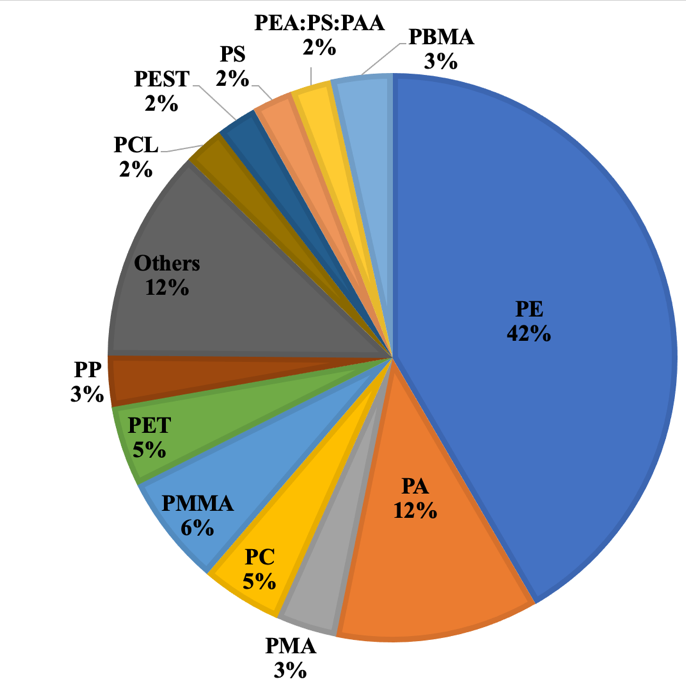  **E** |  |

**Figure 8. Composition of polymers detected in: (A) upper wrack zone (>1 mm); (B) upper wrack zone (<1 mm); (C) lower wrack zone (>1 mm); (D) lower wrack zone (<1 mm); and (E) all samples combined. Polymers were identified using µ-FTIR.** **Note: the percentage of polyamide (PA) may be underestimated as the samples were dried near the glass transition temperature which may affect detection of the polymer by FTIR.**

**Conclusions**

This study focused on quantifying and characterizing MPs in sand collected from a beach near the outlet of Sardis Lake, a major flood-control reservoir in northern Mississippi, as well as assessing pretreatment procedures using MPs generated from naturally weathered plastic. We showed good recoveries for all spiked MPs, except for weathered LDPE film which apparently fragmented during sample preparation. Results for the beach samples showed that the concentration and distribution of MPs on the beach is greatly influenced by dam discharge rates. Concentrations of MPs varied with proximity to the shoreline, with the highest abundance in the wrack zones and the lowest in areas between wrack zones. These finding have implications for targeted sampling of reservoir beaches in future studies. When attempting to compare MPs concentrations between reservoirs, it is imperative to sample from similar zones as well as to account for discharge rates. Additionally, our results suggest that samples should be taken from a variety of points to assess spatial differences, even within a single zone. Sampling is also crucial when it comes to the polymer types being evaluated. The low concentrations of high density polymers seen in this study suggests that they are likely sinking to the bottom of the lake as opposed to washing up on shore. Future studies therefore include lake sediment samples to provide the full picture of MP contamination in the system. As reservoirs are often used as a drinking water source for their communities, it is critical to not only understand the full range of MP contamination in them but also to focus on ways to reduce this pollution. Our findings show that both macro- and micro-plastics accumulate on the wrack zones of reservoir beaches, making them prime locations for plastic cleanup efforts.

**List of abbreviations**

| Polymer | Abbreviation | Polymer | Abbreviation |
| --- | --- | --- | --- |
| Polyamide | PA | Poly (N-methyl acrylamide) | PMA |
| Poly (11-bromoundecyl methacrylate) | PBMA | Polymethyl methacrylate | PMMA |
| Polycarbonate | PC | Polynorbornene | PNB |
| Polycaprolactone | PCL | Polyacrylate:styrene | Polyacrylate:PS |
| Polyethylene | PE | Polyoxymethylene | POM |
| Polyester | PEST | Polypropylene | PP |
| Polyethylene terephthalate | PET | Polystyrene | PS |
| Polyethyl acrylate:styrene:acrylamide | PEA:PS:PAA | Polyurethane | PUR |

**Availability of data and material**

Data is available from the corresponding author upon request.

**Competing interests**

The authors declare no conflict of interest. The funders had no role in the design of the study; in the collection, analyses, or interpretation of data; in the writing of the manuscript, or in the decision to publish the results.

**Funding**

This material is based upon work supported by the U.S. Geological Survey (USGS) under Grant/Cooperative Agreement No. (G16AP00065). The views and conclusions contained in this document are those of the authors and should not be interpreted as representing the opinions or policies of the USGS. Mention of trade names or commercial products does not constitute their endorsement by the USGS.

**Author’s contributions**

J.V.C. conceptualized, supervised, and administered the project; Z.G. led the sampling and measurement campaign; Z.G., K.W., and J.V.C. analyzed and interpreted the data and authored the manuscript; H.L. analyzed select samples by FTIR microscopy. All authors have read and agreed to the published version of the manuscript.

**Acknowledgements**

The authors would like to thank Jimmy Potts at the US Army Corps of Engineers (Vicksburg District) for useful information on Sardis Lake operations. We are also grateful to the Imaging Core in the Department of Biology at the University of Mississippi for access to stereomicroscopes.

**References**

(1) Lee, H.-J.; Song, N.-S.; Kim, J.-S.; Kim, S.-K. Variation and Uncertainty of Microplastics in Commercial Table Salts: Critical Review and Validation. *J. Hazard. Mater.* **2021**, *402*, 123743. https://doi.org/10.1016/j.jhazmat.2020.123743.

(2) Smith, M.; Love, D. C.; Rochman, C. M.; Neff, R. A. Microplastics in Seafood and the Implications for Human Health. *Curr. Environ. Health Rep.* **2018**, *5* (3), 375–386. https://doi.org/10.1007/s40572-018-0206-z.

(3) Wang, J.; Liu, X.; Li, Y.; Powell, T.; Wang, X.; Wang, G.; Zhang, P. Microplastics as Contaminants in the Soil Environment: A Mini-Review. *Sci. Total Environ.* **2019**, *691*, 848–857. https://doi.org/10.1016/j.scitotenv.2019.07.209.

(4) Eerkes-Medrano, D.; Leslie, H. A.; Quinn, B. Microplastics in Drinking Water: A Review and Assessment. *Curr. Opin. Environ. Sci. Health* **2019**, *7*, 69–75. https://doi.org/10.1016/j.coesh.2018.12.001.

(5) Gaston, E.; Woo, M.; Steele, C.; Sukumaran, S.; Anderson, S. Microplastics Differ Between Indoor and Outdoor Air Masses: Insights from Multiple Microscopy Methodologies. *Appl. Spectrosc.* **2020**, *74* (9), 1079–1098. https://doi.org/10.1177/0003702820920652.

(6) Luo, W.; Su, L.; Craig, N. J.; Du, F.; Wu, C.; Shi, H. Comparison of Microplastic Pollution in Different Water Bodies from Urban Creeks to Coastal Waters. *Environ. Pollut.* **2019**, *246*, 174–182. https://doi.org/10.1016/j.envpol.2018.11.081.

(7) K, K. L. D.; Link to external site, this link will open in a new window; Katarina, G.; Krumpen, T.; Thompson, R. C.; Ian, O. Microplastics in Sea Ice and Seawater beneath Ice Floes from the Arctic Ocean. *Sci. Rep. Nat. Publ. Group* **2020**, *10* (1). http://dx.doi.org.umiss.idm.oclc.org/10.1038/s41598-020-61948-6.

(8) Mohamed Nor, N. H.; Kooi, M.; Diepens, N. J.; Koelmans, A. A. Lifetime Accumulation of Microplastic in Children and Adults. *Environ. Sci. Technol.* **2021**, *55* (8), 5084–5096. https://doi.org/10.1021/acs.est.0c07384.

(9) Hartmann, N. B.; Hüffer, T.; Thompson, R. C.; Hassellöv, M.; Verschoor, A.; Daugaard, A. E.; Rist, S.; Karlsson, T.; Brennholt, N.; Cole, M.; Herrling, M. P.; Hess, M. C.; Ivleva, N. P.; Lusher, A. L.; Wagner, M. Are We Speaking the Same Language? Recommendations for a Definition and Categorization Framework for Plastic Debris. *Environ. Sci. Technol.* **2019**, *53* (3), 1039–1047. https://doi.org/10.1021/acs.est.8b05297.

(10) Green, D. S.; Kregting, L.; Boots, B.; Blockley, D. J.; Brickle, P.; da Costa, M.; Crowley, Q. A Comparison of Sampling Methods for Seawater Microplastics and a First Report of the Microplastic Litter in Coastal Waters of Ascension and Falkland Islands. *Mar. Pollut. Bull.* **2018**, *137*, 695–701. https://doi.org/10.1016/j.marpolbul.2018.11.004.

(11) Taha, Z. D.; Md Amin, R.; Anuar, S. T.; Nasser, A. A. A.; Sohaimi, E. S. Microplastics in Seawater and Zooplankton: A Case Study from Terengganu Estuary and Offshore Waters, Malaysia. *Sci. Total Environ.* **2021**, *786*, 147466. https://doi.org/10.1016/j.scitotenv.2021.147466.

(12) Ding, J.; Jiang, F.; Li, J.; Wang, Z.; Sun, C.; Wang, Z.; Fu, L.; Ding, N. X.; He, C. Microplastics in the Coral Reef Systems from Xisha Islands of South China Sea. *Environ. Sci. Technol.* **2019**, *53* (14), 8036–8046. https://doi.org/10.1021/acs.est.9b01452.

(13) Horton, A. A.; Walton, A.; Spurgeon, D. J.; Lahive, E.; Svendsen, C. Microplastics in Freshwater and Terrestrial Environments: Evaluating the Current Understanding to Identify the Knowledge Gaps and Future Research Priorities. *Sci. Total Environ.* **2017**, *586*, 127–141. https://doi.org/10.1016/j.scitotenv.2017.01.190.

(14) Peng, G.; Xu, P.; Zhu, B.; Bai, M.; Li, D. Microplastics in Freshwater River Sediments in Shanghai, China: A Case Study of Risk Assessment in Mega-Cities. *Environ. Pollut.* **2018**, *234*, 448–456. https://doi.org/10.1016/j.envpol.2017.11.034.

(15) Shruti, V. C.; Jonathan, M. P.; Rodriguez-Espinosa, P. F.; Rodríguez-González, F. Microplastics in Freshwater Sediments of Atoyac River Basin, Puebla City, Mexico. *Sci. Total Environ.* **2019**, *654*, 154–163. https://doi.org/10.1016/j.scitotenv.2018.11.054.

(16) Li, J.; Liu, H.; Paul Chen, J. Microplastics in Freshwater Systems: A Review on Occurrence, Environmental Effects, and Methods for Microplastics Detection. *Water Res.* **2018**, *137*, 362–374. https://doi.org/10.1016/j.watres.2017.12.056.

(17) Corcoran, P. L.; Biesinger, M. C.; Grifi, M. Plastics and Beaches: A Degrading Relationship. *Mar. Pollut. Bull.* **2009**, *58* (1), 80–84. https://doi.org/10.1016/j.marpolbul.2008.08.022.

(18) Hwang, J.; Choi, D.; Han, S.; Jung, S. Y.; Choi, J.; Hong, J. Potential Toxicity of Polystyrene Microplastic Particles. *Sci. Rep.* **2020**, *10* (1), 7391. https://doi.org/10.1038/s41598-020-64464-9.

(19) Carson, H. S.; Colbert, S. L.; Kaylor, M. J.; McDermid, K. J. Small Plastic Debris Changes Water Movement and Heat Transfer through Beach Sediments. *Mar. Pollut. Bull.* **2011**, *62* (8), 1708–1713. https://doi.org/10.1016/j.marpolbul.2011.05.032.

(20) Seeley, M. E.; Song, B.; Passie, R.; Hale, R. C. Microplastics Affect Sedimentary Microbial Communities and Nitrogen Cycling. *Nat. Commun.* **2020**, *11* (1), 2372. https://doi.org/10.1038/s41467-020-16235-3.

(21) Rodrigues, A.; Oliver, D. M.; McCarron, A.; Quilliam, R. S. Colonisation of Plastic Pellets (Nurdles) by E. Coli at Public Bathing Beaches. *Mar. Pollut. Bull.* **2019**, *139*, 376–380. https://doi.org/10.1016/j.marpolbul.2019.01.011.

(22) Tiwari, M.; Rathod, T. D.; Ajmal, P. Y.; Bhangare, R. C.; Sahu, S. K. Distribution and Characterization of Microplastics in Beach Sand from Three Different Indian Coastal Environments. *Mar. Pollut. Bull.* **2019**, *140*, 262–273. https://doi.org/10.1016/j.marpolbul.2019.01.055.

(23) Bayo, J.; Rojo, D.; Olmos, S. Abundance, Morphology and Chemical Composition of Microplastics in Sand and Sediments from a Protected Coastal Area: The Mar Menor Lagoon (SE Spain). *Environ. Pollut.* **2019**, *252*, 1357–1366. https://doi.org/10.1016/j.envpol.2019.06.024.

(24) Eo, S.; Hong, S. H.; Song, Y. K.; Lee, J.; Lee, J.; Shim, W. J. Abundance, Composition, and Distribution of Microplastics Larger than 20 μm in Sand Beaches of South Korea. *Environ. Pollut.* **2018**, *238*, 894–902. https://doi.org/10.1016/j.envpol.2018.03.096.

(25) Maynard, I. F. N.; Bortoluzzi, P. C.; Nascimento, L. M.; Madi, R. R.; Cavalcanti, E. B.; Lima, Á. S.; Jeraldo, V. de L. S.; Marques, M. N. Analysis of the Occurrence of Microplastics in Beach Sand on the Brazilian Coast. *Sci. Total Environ.* **2021**, *771*, 144777. https://doi.org/10.1016/j.scitotenv.2020.144777.

(26) Bosker, T.; Guaita, L.; Behrens, P. Microplastic Pollution on Caribbean Beaches in the Lesser Antilles. *Mar. Pollut. Bull.* **2018**, *133*, 442–447. https://doi.org/10.1016/j.marpolbul.2018.05.060.

(27) Minor, E. C.; Lin, R.; Burrows, A.; Cooney, E. M.; Grosshuesch, S.; Lafrancois, B. An Analysis of Microlitter and Microplastics from Lake Superior Beach Sand and Surface-Water. *Sci. Total Environ.* **2020**, *744*, 140824. https://doi.org/10.1016/j.scitotenv.2020.140824.

(28) Liu, S.; Jian, M.; Zhou, L.; Li, W. Distribution and Characteristics of Microplastics in the Sediments of Poyang Lake, China. *Water Sci. Technol.* **2019**, *79* (10), 1868–1877. http://dx.doi.org.umiss.idm.oclc.org/10.2166/wst.2019.185.

(29) Sartain, M.; Wessel, C.; Sparks, E. *Microplastics Sampling and Processing Guidebook*; Mississippi State University: MS, USA, 2018.

(30) Besley, A.; Vijver, M. G.; Behrens, P.; Bosker, T. A Standardized Method for Sampling and Extraction Methods for Quantifying Microplastics in Beach Sand. *Mar. Pollut. Bull.* **2017**, *114* (1), 77–83. https://doi.org/10.1016/j.marpolbul.2016.08.055.

(31) Cole, M.; Webb, H.; Lindeque, P. K.; Fileman, E. S.; Halsband, C.; Galloway, T. S. Isolation of Microplastics in Biota-Rich Seawater Samples and Marine Organisms. *Sci. Rep.* **2014**, *4* (1), 4528. https://doi.org/10.1038/srep04528.

(32) Hurley, R. R.; Lusher, A. L.; Olsen, M.; Nizzetto, L. Validation of a Method for Extracting Microplastics from Complex, Organic-Rich, Environmental Matrices. *Environ. Sci. Technol.* **2018**, *52* (13), 7409–7417. https://doi.org/10.1021/acs.est.8b01517.

(33) Hidalgo-Ruz, V.; Gutow, L.; Thompson, R. C.; Thiel, M. Microplastics in the Marine Environment: A Review of the Methods Used for Identification and Quantification. *Environ. Sci. Technol.* **2012**, *46* (6), 3060–3075. https://doi.org/10.1021/es2031505.

(34) Free, C. M.; Jensen, O. P.; Mason, S. A.; Eriksen, M.; Williamson, N. J.; Boldgiv, B. High-Levels of Microplastic Pollution in a Large, Remote, Mountain Lake. *Mar. Pollut. Bull.* **2014**, *85* (1), 156–163. https://doi.org/10.1016/j.marpolbul.2014.06.001.

(35) Talvitie, J.; Mikola, A.; Koistinen, A.; Setälä, O. Solutions to Microplastic Pollution – Removal of Microplastics from Wastewater Effluent with Advanced Wastewater Treatment Technologies. *Water Res.* **2017**, *123*, 401–407. https://doi.org/10.1016/j.watres.2017.07.005.

(36) Li, X.; Chen, L.; Mei, Q.; Dong, B.; Dai, X.; Ding, G.; Zeng, E. Y. Microplastics in Sewage Sludge from the Wastewater Treatment Plants in China. *Water Res.* **2018**, *142*, 75–85. https://doi.org/10.1016/j.watres.2018.05.034.

(37) Edo, C.; Tamayo-Belda, M.; Martínez-Campos, S.; Martín-Betancor, K.; González-Pleiter, M.; Pulido-Reyes, G.; García-Ruiz, C.; Zapata, F.; Leganés, F.; Fernández-Piñas, F.; Rosal, R. Occurrence and Identification of Microplastics along a Beach in the Biosphere Reserve of Lanzarote. *Mar. Pollut. Bull.* **2019**, *143*, 220–227. https://doi.org/10.1016/j.marpolbul.2019.04.061.

(38) Yu, X.; Ladewig, S.; Bao, S.; Toline, C. A.; Whitmire, S.; Chow, A. T. Occurrence and Distribution of Microplastics at Selected Coastal Sites along the Southeastern United States. *Sci. Total Environ.* **2018**, *613–614*, 298–305. https://doi.org/10.1016/j.scitotenv.2017.09.100.

(39) Aslam, H.; Ali, T.; Mortula, M. M.; Attaelmanan, A. G. Evaluation of Microplastics in Beach Sediments along the Coast of Dubai, UAE. *Mar. Pollut. Bull.* **2020**, *150*, 110739. https://doi.org/10.1016/j.marpolbul.2019.110739.

(40) Scircle, A.; Cizdziel, J. V.; Tisinger, L.; Anumol, T.; Robey, D. Occurrence of Microplastic Pollution at Oyster Reefs and Other Coastal Sites in the Mississippi Sound, USA: Impacts of Freshwater Inflows from Flooding. *Toxics* **2020**, *8* (2). https://doi.org/10.3390/toxics8020035.

(41) Scircle, A.; Cizdziel, J. V.; Missling, K.; Li, L.; Vianello, A. Single-Pot Method for the Collection and Preparation of Natural Water for Microplastic Analyses: Microplastics in the Mississippi River System during and after Historic Flooding. *Environ. Toxicol. Chem.* **2020**, *39* (5), 986–995. https://doi.org/10.1002/etc.4698.

(42) Piñon-Colin, T. de J.; Rodriguez-Jimenez, R.; Pastrana-Corral, M. A.; Rogel-Hernandez, E.; Wakida, F. T. Microplastics on Sandy Beaches of the Baja California Peninsula, Mexico. *Mar. Pollut. Bull.* **2018**, *131*, 63–71. https://doi.org/10.1016/j.marpolbul.2018.03.055.

(43) Dodson, G. Z.; Shotorban, A. K.; Hatcher, P. G.; Waggoner, D. C.; Ghosal, S.; Noffke, N. Microplastic Fragment and Fiber Contamination of Beach Sediments from Selected Sites in Virginia and North Carolina, USA. *Mar. Pollut. Bull.* **2020**, *151*, 110869. https://doi.org/10.1016/j.marpolbul.2019.110869.

(44) Lots, F. A. E.; Behrens, P.; Vijver, M. G.; Horton, A. A.; Bosker, T. A Large-Scale Investigation of Microplastic Contamination: Abundance and Characteristics of Microplastics in European Beach Sediment. *Mar. Pollut. Bull.* **2017**, *123* (1), 219–226. https://doi.org/10.1016/j.marpolbul.2017.08.057.

(45) Carr, S. A.; Liu, J.; Tesoro, A. G. Transport and Fate of Microplastic Particles in Wastewater Treatment Plants. *Water Res.* **2016**, *91*, 174–182. https://doi.org/10.1016/j.watres.2016.01.002.

(46) Raju, S.; Carbery, M.; Kuttykattil, A.; Senthirajah, K.; Lundmark, A.; Rogers, Z.; Scb, S.; Evans, G.; Palanisami, T. Improved Methodology to Determine the Fate and Transport of Microplastics in a Secondary Wastewater Treatment Plant. *Water Res.* **2020**, *173*, 115549. https://doi.org/10.1016/j.watres.2020.115549.

(47) Browne, M. A. Sources and Pathways of Microplastics to Habitats. In *Marine anthropogenic litter*; 2015; pp 229–244.

(48) Sheng, Y.; Liu, Y.; Wang, K.; Cizdziel, J. V.; Wu, Y.; Zhou, Y. Ecotoxicological Effects of Micronized Car Tire Wear Particles and Their Heavy Metals on the Earthworm (Eisenia Fetida) in Soil. *Sci. Total Environ.* **2021**, *793*, 148613. https://doi.org/10.1016/j.scitotenv.2021.148613.

(49) Scherer, C.; Weber, A.; Lambert, S.; Wagner, M. Interactions of Microplastics with Freshwater Biota. In *Freshwater microplastics*; Springer, Cham, 2018; pp 153–180.

(50) Teuten, E. L.; Saquing, J. M.; Knappe, D. R. U.; Barlaz, M. A.; Jonsson, S.; Björn, A.; Rowland, S. J.; Thompson, R. C.; Galloway, T. S.; Yamashita, R.; Ochi, D.; Watanuki, Y.; Moore, C.; Viet, P. H.; Tana, T. S.; Prudente, M.; Boonyatumanond, R.; Zakaria, M. P.; Akkhavong, K.; Ogata, Y.; Hirai, H.; Iwasa, S.; Mizukawa, K.; Hagino, Y.; Imamura, A.; Saha, M.; Takada, H. Transport and Release of Chemicals from Plastics to the Environment and to Wildlife. *Philos. Trans. Biol. Sci.* **2009**, *364* (1526), 2027–2045.

(51) Tourinho, P. S.; Kočí, V.; Loureiro, S.; van Gestel, C. A. M. Partitioning of Chemical Contaminants to Microplastics: Sorption Mechanisms, Environmental Distribution and Effects on Toxicity and Bioaccumulation. *Environ. Pollut.* **2019**, *252*, 1246–1256. https://doi.org/10.1016/j.envpol.2019.06.030.

(52) Suaria, G.; Perold, V.; Lee, J. R.; Lebouard, F.; Aliani, S.; Ryan, P. G. Floating Macro- and Microplastics around the Southern Ocean: Results from the Antarctic Circumnavigation Expedition. *Environ. Int.* **2020**, *136*.

(53) Ziajahromi, S.; Neale, P. A.; Rintoul, L.; Leusch, F. D. L. Wastewater Treatment Plants as a Pathway for Microplastics: Development of a New Approach to Sample Wastewater-Based Microplastics. *Water Res.* **2017**, *112*, 93–99. https://doi.org/10.1016/j.watres.2017.01.042.

(54) Ory, N. C.; Sobral, P.; Ferreira, J. L.; Thiel, M. Amberstripe Scad Decapterus Muroadsi (Carangidae) Fish Ingest Blue Microplastics Resembling Their Copepod Prey along the Coast of Rapa Nui (Easter Island) in the South Pacific Subtropical Gyre. *Sci. Total Environ.* **2017**, *586*, 430–437. https://doi.org/10.1016/j.scitotenv.2017.01.175.

(55) Martí, E.; Martin, C.; Galli, M.; Echevarría, F.; Duarte, C. M.; Cózar, A. The Colors of the Ocean Plastics. *Environ. Sci. Technol.* **2020**, *54* (11), 6594–6601. https://doi.org/10.1021/acs.est.9b06400.

(56) Rochman, C. M.; Brookson, C.; Bikker, J.; Djuric, N.; Earn, A.; Bucci, K.; Athey, S.; Huntington, A.; McIlwraith, H.; Munno, K.; Frond, H. D.; Kolomijeca, A.; Erdle, L.; Grbic, J.; Bayoumi, M.; Borrelle, S. B.; Wu, T.; Santoro, S.; Werbowski, L. M.; Zhu, X.; Giles, R. K.; Hamilton, B. M.; Thaysen, C.; Kaura, A.; Klasios, N.; Ead, L.; Kim, J.; Sherlock, C.; Ho, A.; Hung, C. Rethinking Microplastics as a Diverse Contaminant Suite. *Environ. Toxicol. Chem.* **2019**, *38* (4), 703–711. https://doi.org/10.1002/etc.4371.

(57) Bretas Alvim, C.; Bes-Piá, M. A.; Mendoza-Roca, J. A. Separation and Identification of Microplastics from Primary and Secondary Effluents and Activated Sludge from Wastewater Treatment Plants. *Chem. Eng. J.* **2020**, *402*, 126293. https://doi.org/10.1016/j.cej.2020.126293.

(58) Bissen, R.; Chawchai, S. Microplastics on Beaches along the Eastern Gulf of Thailand – A Preliminary Study. *Mar. Pollut. Bull.* **2020**, *157*, 111345. https://doi.org/10.1016/j.marpolbul.2020.111345.

(59) Gewert, B.; M. Plassmann, M.; MacLeod, M. Pathways for Degradation of Plastic Polymers Floating in the Marine Environment. *Environ. Sci. Process. Impacts* **2015**, *17* (9), 1513–1521. https://doi.org/10.1039/C5EM00207A.

(60) Doyen, P., Hermabessiere, L., Dehaut, A., Himber, C., Decodts, M., Degraeve, T., Delord, L., Gaboriaud, M., Moné, P., Sacco, J. and Tavernier, E., 2019. Occurrence and identification of microplastics in beach sediments from the Hauts-de-France region. *Environ. Sci. Pollut. Res.***2019**, *26*(27), 28010-28021. https://doi.org/10.1007/s11356-019-06027-8.

**Supplemental Information**

**Distribution and characteristics of microplastics in beach sand near the outlet of a major reservoir in north Mississippi, USA**

**Table S1. Microplastics (1-5 mm size fraction) abundances and morphologies in the beach sand. No microplastics in this size class were found in-between the wrack zones.**

| Locations | | Average abundance (± 1SD, n=3) | | Morphologies (%) | | | |
| --- | --- | --- | --- | --- | --- | --- | --- |
|  |  | Particles/kg | Particles/m^2^ | Fibers | Fragments | Beads | Films |
| Lower wrack zone | Site 1 | 77 ± 40 | 3300 ± 1700 | 76.5 | 14.7 | 0.0 | 8.8 |
|  | Site 2 | 40 ± 23 | 1700 ± 1000 | 65.2 | 17.4 | 0.0 | 17.4 |
|  | Site 3 | 37 ± 31 | 1600 ± 1300 | 81.8 | 9.1 | 0.0 | 9.1 |
|  | Site 4 | 50 ± 42 | 2200 ± 1800 | 75.0 | 8.3 | 0.0 | 16.7 |
|  | Site 5 | 67 ± 25 | 2900 ± 1100 | 71.0 | 16.1 | 0.0 | 12.9 |
| Upper wrack zone | Site 6 | 12 ± 12 | 500 ± 500 | 85.7 | 14.3 | 0.0 | 0.0 |
|  | Site 7 | 40 ± 31 | 1700 ± 1300 | 79.2 | 4.2 | 0.0 | 16.7 |
|  | Site 8 | 23 ± 26 | 1000 ± 1100 | 88.9 | 11.1 | 0.0 | 0.0 |
|  | Site 9 | 50 ± 35 | 2200 ± 1500 | 84.6 | 7.7 | 0.0 | 7.7 |
|  | Site 10 | 19 ± 17 | 820 ± 740 | 80.0 | 6.7 | 0.0 | 13.3 |

**Table S2. Microplastics (<1 mm size fraction) abundances and morphologies in the beach sand.**

| Locations | | Average abundance (± 1SD, n=3) | | | Morphologies (%) | | | |
| --- | --- | --- | --- | --- | --- | --- | --- | --- |
|  |  | Particles/kg | Particles/m^2^ | | Fibers | Fragments | Beads | Films |
| Lower wrack zone | Site 1 | 1070 ± 210 | | 46000 ± 9100 | 70.6 | 20.7 | 3.4 | 5.3 |
|  | Site 2 | 730 ± 37 | | 31000 ± 1600 | 54.3 | 31.1 | 4.7 | 9.8 |
|  | Site 3 | 640 ± 57 | | 28000 ± 2500 | 64.4 | 21.5 | 7.4 | 6.7 |
|  | Site 4 | 760 ± 70 | | 33000 ± 3000 | 57.7 | 27.3 | 7.5 | 7.5 |
|  | Site 5 | 1400 ± 190 | | 58000 ± 8100 | 71.6 | 18.8 | 7.5 | 2.1 |
| Upper wrack zone | Site 6 | 470 ± 98 | | 21000 ± 4300 | 57.9 | 22.5 | 13.5 | 6.2 |
|  | Site 7 | 470 ± 77 | | 20000 ± 3300 | 56.3 | 27.3 | 10.2 | 6.3 |
|  | Site 8 | 600 ± 140 | | 26000 ± 6100 | 50.9 | 29.2 | 13.4 | 6.5 |
|  | Site 9 | 480 ± 55 | | 20800 ± 2400 | 61.7 | 16.7 | 14.4 | 7.2 |
|  | Site 10 | 650 ± 3 | | 28000 ± 140 | 71.1 | 22.2 | 5.2 | 1.5 |
| In-between zone | Site 11 | 250 ± 100 | | 10800 ± 4500 | 56.0 | 28.0 | 2.7 | 13.3 |
|  | Site 12 | 270 ± 57 | | 12000 ± 2500 | 48.8 | 32.5 | 11.3 | 7.5 |
|  | Site 13 | 180 ± 29 | | 7900 ± 1300 | 52.7 | 36.4 | 3.6 | 7.3 |
|  | Site 14 | 370 ± 19 | | 16000 ± 800 | 53.6 | 33.0 | 7.1 | 6.3 |
|  | Site 15 | 280 ± 28 | | 12000 ± 1200 | 43.4 | 32.5 | 19.3 | 4.8 |


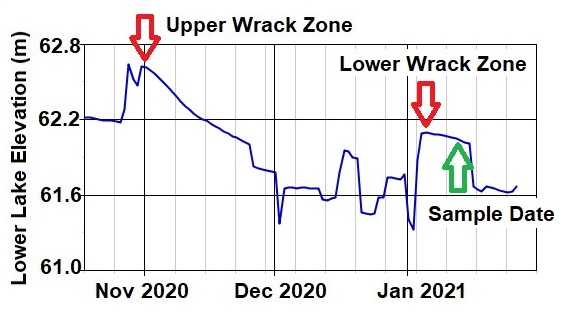


**Figure S1. USGS river gauge data showing the elevation of lower Sardis Lake where the beach is situated, as well as the sampling date (green arrow) and the elevation and date the two wrack zones were formed (red arrows). The lake discharge rates corresponding to the upper and lower wrack zones were ~160 m^3^/sec and ~125 m^3^/sec, respectively.**

| **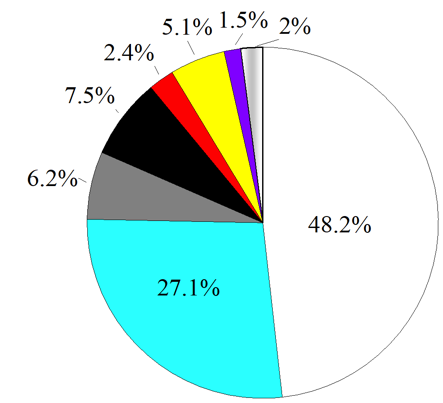** | **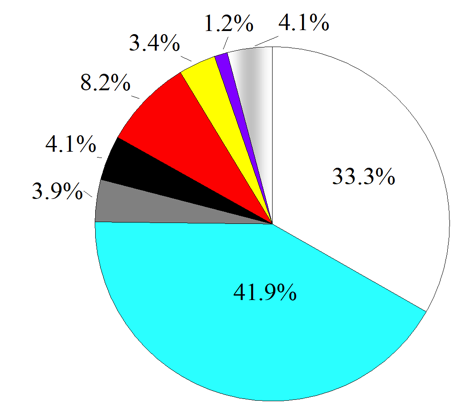** | **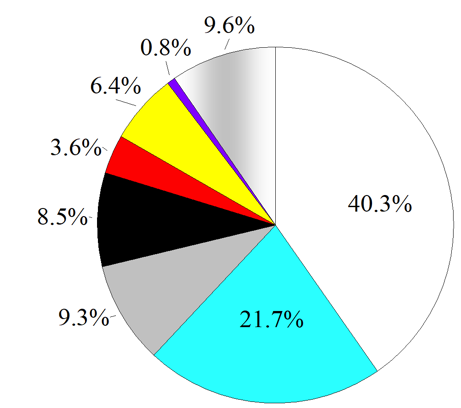** | **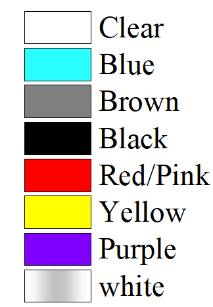** |
| --- | --- | --- | --- |

**Figure S2. Color distribution of small MPs extracted from lower wrack zone (left), in-between zone (middle) and upper wrack zone (right).**
